# Aristolochic acid I determine the phenotype and activation of macrophages in acute and chronic kidney disease

**DOI:** 10.1038/s41598-018-30628-x

**Published:** 2018-08-15

**Authors:** Mohsen Honarpisheh, Orestes Foresto-Neto, Stefanie Steiger, Franziska Kraft, Paulina Koehler, Ekaterina von Rauchhaupt, Jan Potempa, Karina Adamowicz, Joanna Koziel, Maciej Lech

**Affiliations:** 1 Klinikum der Ludwig-Maximilians-Universität München, Medizinische Klinik und Poliklinik IV, Department of Nephrology, LMU Munich, Germany; 2 Departments of Microbiology, Faculty of Biochemistry, Biophysics and Biotechnology, Jagiellonian University, Krakow, Poland

## Abstract

Acute and chronic kidney injuries are multifactorial traits that involve various risk factors. Experimental animal models are crucial to unravel important aspects of injury and its pathophysiological mechanisms. Translating knowledge obtained from experimental approaches into clinically useful information is difficult; therefore, significant attention needs to be paid to experimental procedures that mimic human disease. Herein, we compared aristolochic acid I (AAI) acute and chronic kidney injury model with unilateral ischemic-reperfusion injury (uIRI), cisplatin (CP)- or folic acid (FA)-induced renal damage. The administration of AAI showed significant changes in serum creatinine and BUN upon CKD. The number of neutrophils and macrophages were highly increased as well as AAI-induced CKD characterized by loss of tubular epithelial cells and fibrosis. The *in vitro* and *in vivo* data indicated that macrophages play an important role in the pathogenesis of AA-induced nephropathy (AAN) associated with an excessive macrophage accumulation and an alternative activated macrophage phenotype. Taken together, we conclude that AA-induced injury represents a suitable and relatively easy model to induce acute and chronic kidney injury. Moreover, our data indicate that this model is appropriate and superior to study detailed questions associated with renal macrophage phenotypes.

## Introduction

Acute kidney injury (AKI) is a global pathologic condition that occurs at a high incidence in hospitalized individuals with acute illness^1,2^ associated with increased mortality^3,4^. AKI is characterized by tubular injury that may be replaced by new proliferating tubular epithelial cells. This process of complete recovery from AKI is still being a topic of speculation. Various studies showed that AKI is associated with increased incidence of chronic kidney disease (CKD) due to uncontrolled and unbalanced repair processes that may lead to the development of fibrosis^5^. Processes such as moderate inflammation are necessary for the initiation of tissue repair. On the other hand, persistent inflammatory responses result in loss of kidney function and disease progression^6^. Several experimental animal models of acute and chronic kidney disease mimic the human disease. Some animal models involve operative manipulation; others are induced by intervention with toxic substances that affect renal tissue. An appropriate animal model is crucial in order to prove scientific hypothesis or evaluate a new therapeutic approach. One of the best-characterized kidney injury models is ischemia reperfusion-induced injury (IRI). Ischemic damage is associated with impairment of oxygen supply and nutrient delivery to renal tissue, as well as accumulation of metabolic waste products in renal cells. As a result of injury, tubular epithelial cells die leading to further activation of innate immune responses^7,8^. Not only antigen presenting cells but also tubular epithelial cells were shown to participate in inflammatory responses upon activation of evolutionarily conserved pattern recognition receptors (PRRs), which detect endogenous ligands released during inflammatory cell death^9^. Clinically, ischemia is a leading cause of AKI and it may result from the poor cardiac condition or kidney transplantation^10^. Both bilateral renal ischemia reperfusion (bIR) and unilateral renal ischemia-reperfusion (uIR) are commonly used as experimental models of kidney injury. In contrast to *in vivo* models, *in vitro* experiments display many limitations^11^. Reports demonstrated that experiments with isolated renal tubules or perfused kidneys rather focus on oxidative stress than on ischemic conditions, which are difficult to achieve without affecting the environmental conditions in cell culture^12,13^. Unlike surgically induced models, chemically induced models are often more reliable for mimicking some of the features of the disease in *in vitro* approaches. They are often easy to perform and yet clinically relevant^14^. For example, aristolochic acid I (AAI), which is present in various herbal remedies, is associated with the development of nephropathies such as Chinese-herb nephropathy (CHN) and Balkan endemic nephropathy (BEN). Aristolochic acid nephropathy (AAN) is characterized by acute tubular necrosis, tubular atrophy, lymphocytic infiltrates and renal fibrosis^15^. AAs are well known for their nephrotoxic effects in rodents^16,17^. Moreover, various experimental studies described two distinct phases of AAI-induced damage. An early acute phase up to day 5, which is characterized by acute tubular epithelial cell necrosis and a chronic phase that is associated with interstitial cell infiltration leading progressively to tubular atrophy and renal fibrosis^18^. Another nephrotoxic compound is cisplatin (cis- diamminedichloroplatinum (II), CDDP), an antineoplastic drug widely prescribed for the treatment of solid-organ cancers and its various side effects include nephrotoxicity^19^. Cisplatin is believed to mediate its cytotoxic effects through its binding to DNA and the formation of cross-links between DNA-strands, which affects replication processes^20^, in particular affecting mitochondrial DNA. Cisplatin-induced cell death correlates with the density of mitochondria, and depletion of mitochondrial DNA makes immune cells highly resistant to cisplatin^21^. This could explain the sensitivity of renal proximal tubular cells, which show high mitochondrial density to cisplatin-induced damage^22,23^. Additionally, mitochondrial DNA possesses less efficient DNA repair mechanisms than nuclear DNA^24^. *In vitro*, cisplatin causes apoptotic and necrotic cell death, which occurs in a dose-dependent manner^25,26^. Unfortunately, i*n vivo* administration of cisplatin is often associated with increased mortality due to necrosis and apoptosis as well as various pro-inflammatory responses in the kidney^27,28^, and^29^. Another well-known chemical used to induce acute kidney injury is folic acid (FA). Although it is recognized as an essential nutrient in humans, a high dose of FA can cause acute toxicity in mice. Acute tubular necrosis and reduced glomerular filtration upon administration of this nephrotoxic substance can be observed in various mouse strains. Upon injection, FA will be filtered first through the glomerulus and then precipitates in the tubules leading to tubular damage with loss of epithelial cell integrity^30,31^. Despite regenerative changes, incomplete healing, leucocyte infiltration and interstitial fibrosis can be observed^32,33^.

All these models are widely used for experimental and therapeutic approaches. Previously, beneficial features or limitation of these models were however not depicted and characterized in comparative studies. Herein, we evaluated histological parameters and profiles of infiltrating immune cells as well as relevant transcriptomic changes in selected animal models. We also modified the models to avoid mortality and investigated both the acute and chronic injury phase evidenced by tubular loss and fibrosis. Moreover, we pick out AAI-induced injury as a most promising approach and used human renal proximal tubular cells and macrophages to study AAI-induced injury in *in vitro* model.

## Materials and Methods

### Mice

Female, 8-week-old C57BL/6 mice were randomly assigned to study groups (n = 6–10 per each group). The animals were either intraperitoneally injected with a solution of AA-I salt in PBS (5 mg/kg body weight) on day 0, 2, 4, 6, 8, 10 (AA), or with folic acid as single injection (250 mg/kg body weight) on day 1 (FA), or with cisplatin as single injection (5 mg/kg body weight) on day 1 (CP) or with the solvent (control/vehicle). For ischemia-reperfusion injury (IRI) mice were anesthetized prior to sham surgery or unilateral renal pedicle clamping with a microaneurysm clamp (Medicon, Germany) via a flank incision (ischemia time was 35 minutes). All animals were sacrificed on day 3 (acute injury) or day 30 (chronic injury) of the experiment. A control/vehicle/sham operated groups did not receive any intervention and did not show any pathological changes within a kidney or differences in gene expression. Therefore the control groups were serving as a comparison group (referred as vehicle) when treatment results were evaluated. Animal experiments were performed in accordance with the European law regarding protection of animal welfare and with approval by the local government authorities, Regierung von Oberbayern or II LKE in Krakow (reference number: 55.2-1-54-2532-189-2015, 55.2-1-54-2532.0-70-2016, 55.2-1-54-2532-63-12, 70-2018).

### Histological analysis

Kidney tissues were fixed in 4% paraformaldehyde and embedded in paraffin. The 2–4 µm sections were stained for acid–Schiff (PAS) and immunostaining. Tubular injury, immune cell infiltration, and fibrosis were scored by assessing the percentage of immune cells, stained area in a semi-quantitative manner or quantified with Image J or Photoshop software and normalized to whole kidney section area. For each kidney at least 5 high power fields were analyzed. Neutrophils were detected by immunostaining using rat anti-mouse Ly-6B.2 (Serotec, UK), macrophages were detected with F4/80 stain (Serotec, Oxford, UK, 1:50) and renal fibrosis was visualized by Masson’s trichrome or α-SMA staining (Dako, Glostrup, Denmark, 1:100).

### RNA extraction, reverse transcription and qRT-PCR

Pure Link RNA Mini Kit (Ambion, Germany) was used to extract total RNA from renal tissue stored in RNA later, according to the manufacturer’s instructions. cDNA was synthesized from 1 µg of total RNA by reverse transcription polymerase chain reaction (PCR) using Superscript II reverse transcriptase (Thermo Fisher, Germany) according to manufacturer instructions. Quantitative real-time PCR (qRT-PCR) from cDNA was performed a Light Cycler 480 (Roche, Germany). 18 s rRNA was used as a reference transcript for relative quantification. Controls consisting of ddH2O were negative for targets and reference genes. The melting curve profiles were analyzed. Amplicons were visualized on agarose gels to evaluate for unspecific products. All primers used for amplification were purchased from Metabion (Martinsried, Germany):

Kim-1: Fw-TCAGCTCGGGAATGCACAA; Rv-TGGTTGCCTTCCGTGTCTCT;

NGAL: Fw- AATGTCACCTCCATCCTGGT; Rv-ATTTCCCAGAGTGAACTGGC;

Cxcl2: Fw-CGGTCAAAAAGTTTGCCTTG; Rv-TCCAGGTCAGTTAGCCTTGC;

Ccl2: Fw- CCTGCTGTTCACAGTTGCC; Rv-ATTGGGATCATCTTGCTGGT;

IL-10: Fw-ATCGATTTCTCCCCTGTGAA; Rv-TGTCAAATTCATTCATGGCCT;

IL-6:Fw-TGATGCACTTGCAGAAAACA;Rv-ACCAGAGGAAATTTTCAATAGGC;

TNF-α: Fw- ATGGGCTACAGGCTTGTCACTC; Rv-CTCTTCTGCCTGCTGCACTTTG;

Bax: Fw-TTGCTGATGGCAACTTCAAC; Rv-GATCAGCTCGGGCACTTTAG;

Bcl2: Fw-CCTGTCGCAGTTGGGTTC; Rv-TGAAGTGCAGTTCTACCCAGG;

IRF4: Fw-TGCAAGCTCTTTGACACACA; Rv-CAAAGCACAGAGTCACCTGG;

IRF5: Fw-ATGGGGACAACACCATCTTC; Rv-CAGGTTGGCCTTCCACTTG;

IRAK-M: Fw-CACTGCTGGGAGAGCTTTG; Rv-CCAGCCAGCTGTTTGAAAGT;

TGF-β: Fw-GGAGAGCCCTGGATACCAAC; Rv-CAACCCAGGTCCTTCCTAAA;

IL-13: Fw- CAGCCTCCCCGATACCAAAA; Rv-TCCTCATTAGAAGGGGCCGT;

CTGF: Fw-AGCTGACCTGGAGGAAAACA; Rv-CCGCAGAACTTAGCCCTGTA;

E-cadherin:Fw-GAGGTCTACACCTTCCCGGT; Rv-CCACTTTGAATCGGGAGTCT;

FSP-1: Fw-CAGCACTTCCTCTCTCTTGG; Rv-TTTGTGGAAGGTGGACACAA;

α-SMA: Fw-ACTGGGACGACATGGAAAAG; Rv-GTTCAGTGGTGCCTCTGTCA.

### Renal functional parameter

Serum creatinine levels were measured using the Jaffé method and blood urea nitrogen (BUN) was measured using an enzymatic test, according to the manufacturer’s instructions (Diasys, Germany).

### Air pouch mouse model and apoptotic neutrophils

C57BL/6 N wildtype mice were injected with 3 ml of sterile air subcutaneously (s.c.) into the back as previously described^34^. After two days, 5 mg monosodium urate crystals (InvivoGen) in 1 ml PBS were injected into the air pouches. The pouch fluids, which mainly consist of neutrophils, were harvested using cold PBS after 12 hours. Cells from the air pouch fluids were then stained with the cell tracker CM-Dil fluorescent dye (ThermoFisher Scientific) for 30 minutes. To induce apoptosis of neutrophils (used for macrophage phagocytosis assay), CM-Dil stained cells were treated with 10 ng/ml TNFα (ImmunoTools) and 2.5 ng/ml phorbol myristate acetate (PMA, Sigma) for 2 hours at 37 °C^35^.

### *In vitro* cell experiments

Isolation and culture of primary mouse bone marrow myeloid cells and their differentiation into macrophages was carried out on 10 cm dishes for 7 days in RPMI medium containing 10 ng/mL recombinant mouse macrophage colony stimulating factor (rmM-CSF) at 37 °C and 5% CO_2_. Fresh medium was added on day 2 and changed on day 5 respectively. On day 7 cells were detached using trypsin. 250.000 cells were seeded per well in 6-well plate. 10.000 cells were used for 96 well plates. Medium was replaced by plain RPMI before exposure to AAI. For acute phase 10 µM and 50 µM AAI were used. For chronic phase the stimulation with fresh medium containing 10 µM AAI was carried out on every alternate day for six days (overall 3 stimulations). Mouse tubular cells (MTC) were cultured in DMEM medium (10% FCS, 1%PS). AAI, ≥97% salt was purchased from Sigma (Taufkirchen, Germany). Stock solutions (10 mM and 500 µM) were prepared in PBS. Proliferating cell cultures were exposed to nominal concentrations of 1–1000 µM AAI for 2, 4, 24 and 48 hours. For cytotoxicity and cell death *in vitro* lactate dehydrogenase (LDH) release and propidium iodide (PI) red-fluorescent nuclear were used. The LDH assay (Roche, Germany) was designed according to the manufacturer’s instructions. The PI (Cayman, USA) fluorescence signals were detected using a Leica fluorescence microscope and quantified using ImageJ.

### FACS analysis

Flow cytometry was performed with either BD FACSCanto II or BDCalibur, and the data analyzed using FlowJo software. AAI-treated macrophages were stained for AnnexinV and PI to distinguish between live (AnV- PI-), apoptotic (AnV+ PI−) and late apoptotic/primary necrotic (AnV + PI + ) cells. For phagocytosis assays, bone marrow-derived M-CSF differentiated macrophages were first treated with 50 µM AAI and then co-cultured with CM-Dil-stained apoptotic neutrophils for 2 hours. After incubation, cells were collected and stained with the surface marker anti-mouse CD11b-BV510 (BioLegend), F4/80-APC (BioRad) and Ly6G-PE/Cy7 (BioLegend). F4/80+ CD11b+ macrophages, which were also positive for CM-Dil+ Ly6G+, were identified as macrophages that have phagocytized apoptotic neutrophils. For intracellular reactive oxygen species (ROS) production in macrophages, M-CSF-differentiated macrophages were stimulated with 50 µM AAI for 2 and 4 hours. After stimulation, macrophages were incubated with the general oxidative stress indicator CM-H2DCFDA (1 µM, Molecular Probes) at 37 °C for 30 minutes, washed and resuspended in warm PBS for flow cytometry analysis. For Macrophage differentiation the stimulation with fresh medium containing 10 µM AAI was carried out on every alternate day for six days (overall 3 stimulations). For flow cytometry analysis kidneys from control, acute and chronic groups were washed in cold PBS, de-capsulated and subjected to single cells suspension by sieve them and incubate in collagen type-I for 30 min. The single cells suspensions were washed in cold PBS and re-suspend in 300 µl of FACS buffer. The cells were stained with anti-mouse macrophage/myeloid cells markers CD11b-V450 (BD Bioscience), CD45-PE/Cy5, F4/80-APC, CD206-FITC, Cx3CR1-PE (Bio-Legend), anti-mouse CD11b-BV510 (BD bioscience), CD45-PE/Cy5, CD3-PE, CD4-PacificBlue, CD8-APC/Cy7, Ly6G-PE/Cy7 (BioLegend) were used to identify T cells populations and neutrophils.

### Statistical analysis

Values are expressed as the means ± standard error of the mean (SEM). One-way ANOVA analysis of variance were conducted to determine significance, Tukey’s multiple comparison test was used for all-possible pairwise comparisons (control versus multiple treatment or stimulation groups), t-test was used for non-multiple testing, where only two groups were compared with each other. GraphPad Prism software was used for the calculations.

## Results

### Induction of kidney injury with ischemia-reperfusion, AA, FA and cisplatin

In order to examine the pathogenesis of acute and chronic kidney disease, we established various mouse models of renal injury using IRI, AAI, FA or CP. To explore the nature of renal inflammation caused by different chemical agents or ischemic injury, renal damage was induced and explored at different time points, as illustrated in Supplementary Fig. 1A. The observed changes in body weight were more prominent in AA- and cisplatin-induced kidney injury compared to ischemic and FA-induced kidney damage on day 3 and day 30 respectively (Supplementary Fig. 1B,C).

### Differences between the selected acute kidney injury models in C57BL/6 mice

**Figure 1:**
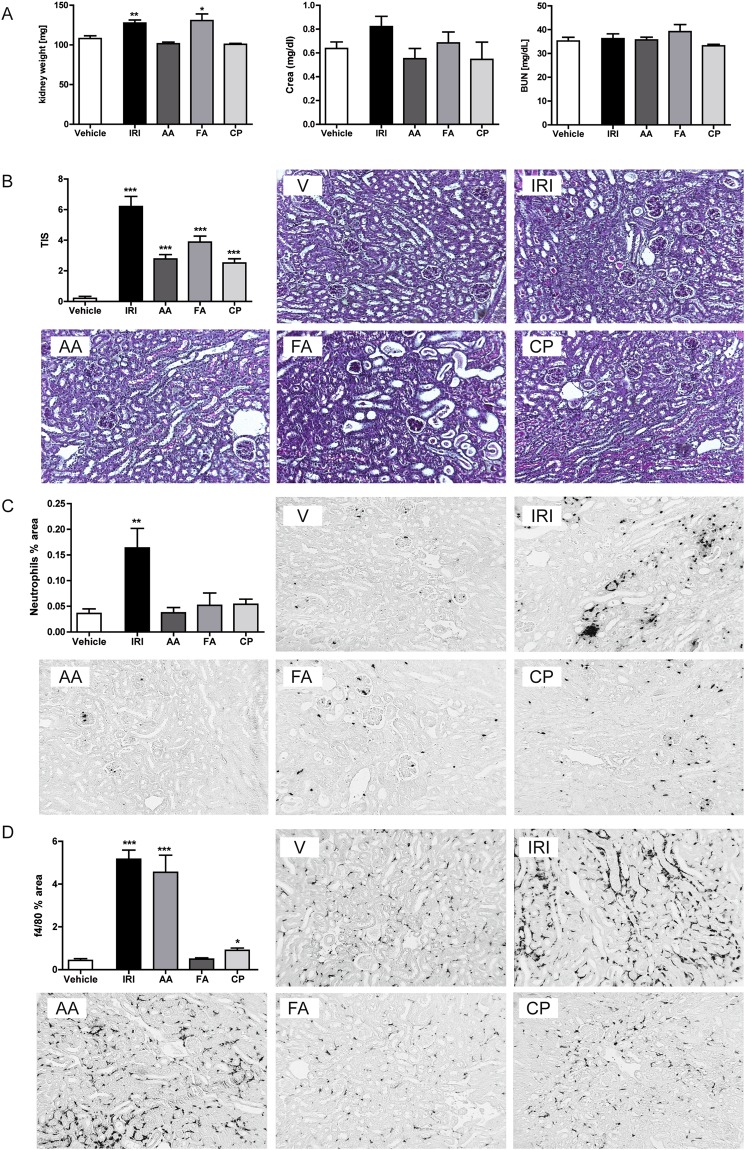
Induction of acute kidney injury. (**A**) Kidney weight, creatinine, and BUN; kidney injury score and infiltration of inflammatory cells, wildtype mice underwent surgery or intervention and kidneys were harvested 3 days later. Renal sections were stained with PAS (**B**), neutrophils (**C**) and F4/80 (**D**) Images illustrate representative sections. The indices for cell infiltration or fibrosis were determined by quantitative morphometry as described in the methods section. Values represent means ± SEM from 30 high power fields of investigated kidneys from 6–10 mice in each group. Data are expressed as means ± SEM. *p < 0.05, **p < 0.01, ***p < 0.001 versus control animal group of the same time point.

**Figure 2:**
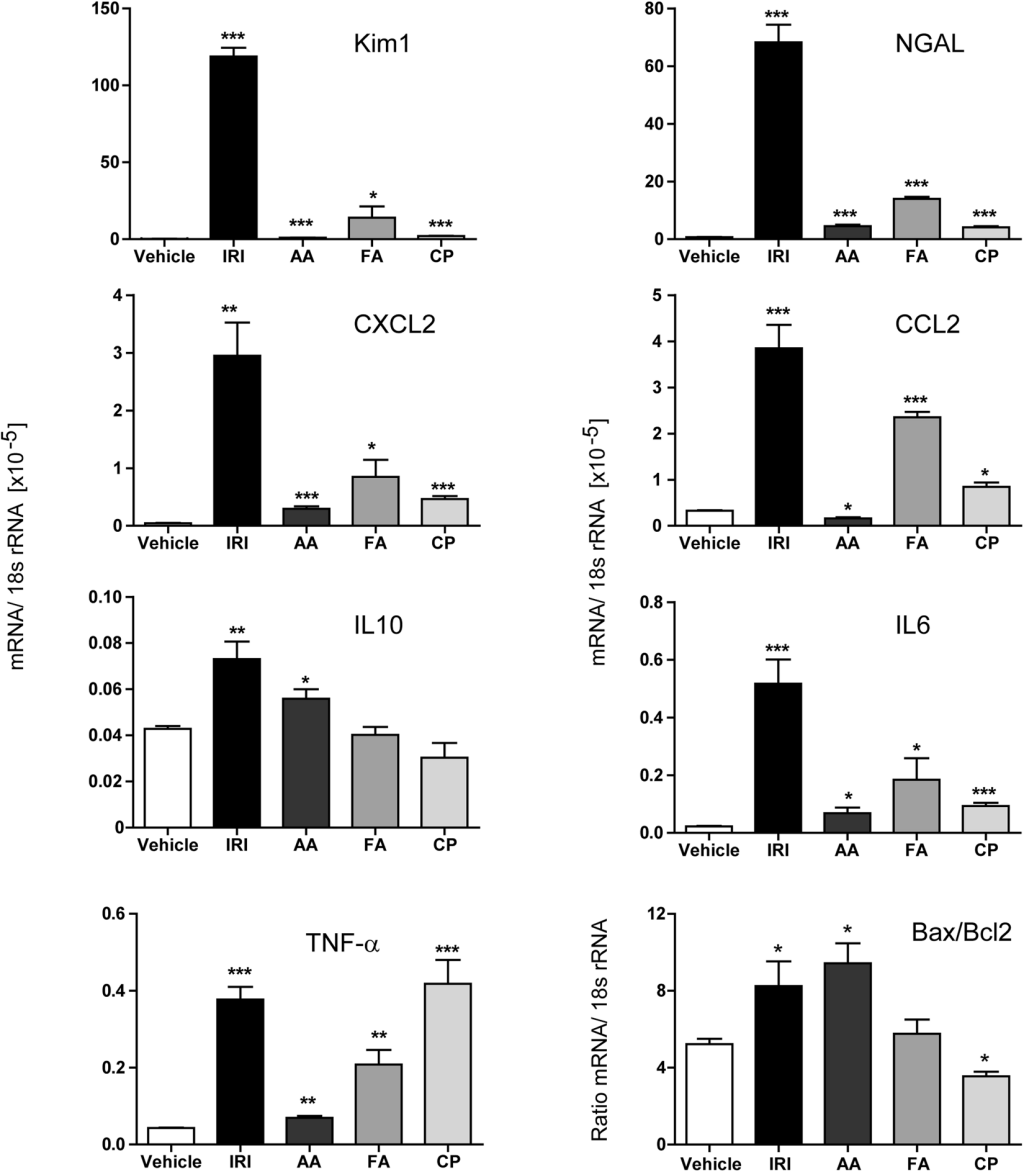
Induction of acute kidney injury. mRNA expression levels were determined by real-time RT-PCR and related to the respective 18 s rRNA expression. Data are expressed as means ± SEM. *p < 0.05, **p < 0.01, ***p < 0.001, versus control animal group of the same time point.

In all models, the serum creatinine and BUN levels did not significantly increase during the acute injury phase indicating a yet unaffected kidney function (Fig. 1A). Consistently, we did also not observe any reduced mobility or increased mortality. Further, we determined histological changes by PAS, neutrophil, CD3+ lymphocytes, CD45+ cells and F4/80 macrophage staining. The results in Fig. 1B illustrate that kidney histology of the vehicle (V) group showed normal appearance and that all experimental groups developed significant tubulointerstitial damage including tubular dilatation and atrophy 3 days upon injury, as quantified and illustrated in Fig. 1B. Excessive infiltration of neutrophils and increase in lymphocytes number was observed exclusively in mice that underwent ischemic injury (Fig. 1C and Supplementary Fig. 2). Moreover, ischemic mice and mice treated with AA exhibited more than 10 times increased levels of F4/80+ cells compared to vehicle-treated animals. To determine whether all models induce inflammation in a similar extent during the acute phase of kidney injury, mRNA expression of pro-inflammatory cytokines and renal damage markers were measured in total kidney tissue (Fig. 2). The expression pattern indicated that IRI induces the most prominent injury, which correlated with the prominent renal damage and F4/80+ macrophage infiltration. Together, all tested experimental injury models are suitable for investigating acute renal pathology. The ischemic injury and the AAN model display the strongest infiltration of macrophages.

### Pronounced differences between acute vs. chronic kidney injury models

**Figure 3:**
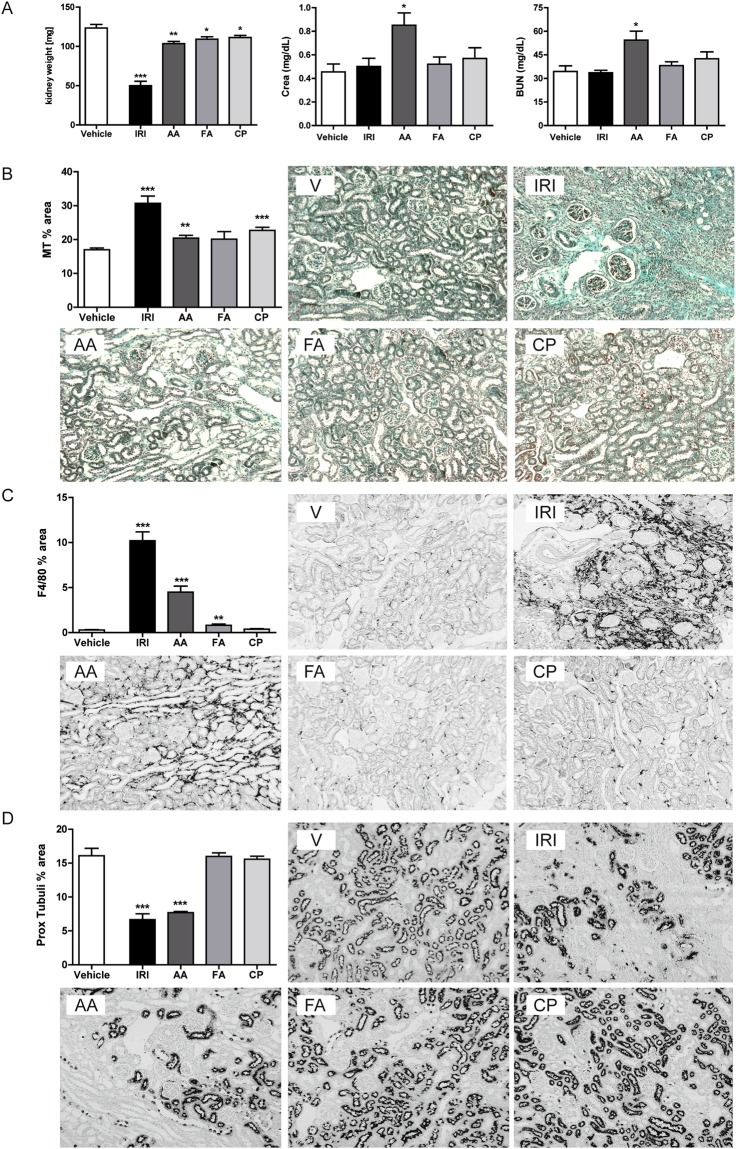
Induction of chronic kidney injury. (**A**) Kidney weight, creatinine, and BUN; kidney injury score and infiltration of inflammatory cells, wild type mice underwent surgery or intervention and kidneys were harvested 30 days later. Renal sections were stained with Masson’s trichrome (MT) (**B**), F4/80 (**C**) and proximal tubules (**D**). Images illustrate representative sections. The indices for cell infiltration or fibrosis were determined by quantitative morphometry as described in the methods section. Values represent means ± SEM from 30 high power fields of investigated kidneys from 6–10 mice in each group. Data are expressed as means ± SEM. *p < 0.05, **p < 0.01, ***p < 0.001, versus control animal group of the same time point.

**Figure 4:**
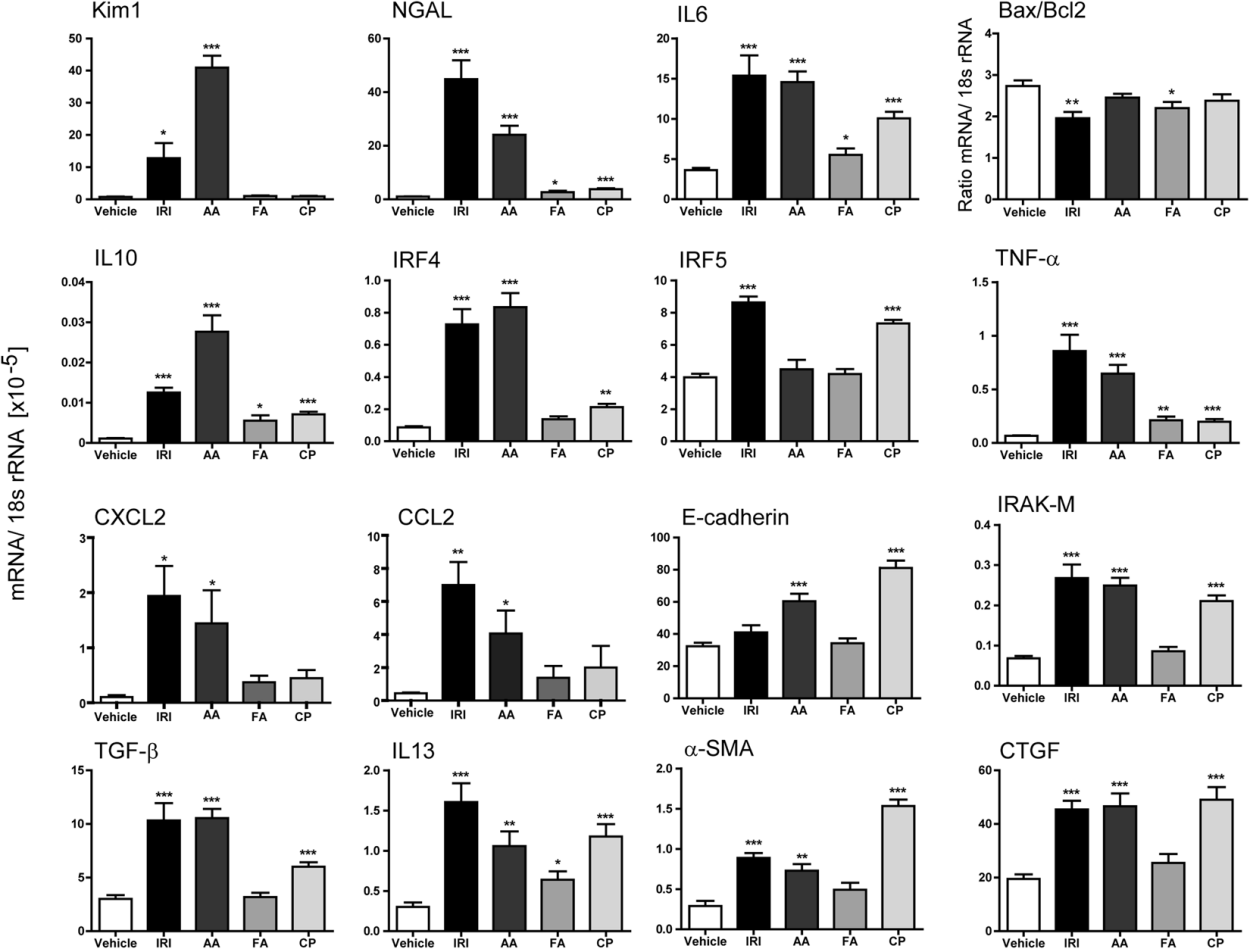
Induction of chronic kidney injury. (**A**) mRNA expression levels were determined by real-time RT-PCR and related to the respective 18 s rRNA expression. Data are expressed as means ± SEM. *p < 0.05, **p < 0.01, ***p < 0.001, versus control animal group of the same time point.

Since many aspects of kidney disease are considered as chronic, and disease progression is a crucial aspect of many investigations, a robust mouse model of CKD is required. After inducing acute injury mice were followed for additional days in order to evaluate chronic inflammation, fibrosis or regeneration of renal tissue. None of the herein tested procedures led to a higher rate of mortality in mice. Although mice with AAI-induced CKD did not increase their body weight within 30 days compared to the other injury models (Supplementary Fig. 1C). During chronic inflammation, all experimental groups developed features of CKD as indicated by changes in kidney weight, immune cell infiltration, and renal fibrosis after 30 days (Fig. 3A–D and Supplementary Fig. 2). However, only mice with AA-induced nephropathy presented with significantly increased serum creatinine and BUN levels indicating renal impairment (Fig. 3A). Unilateral ischemic injury does not lead to significant changes in renal parameters despite the presence of fibrosis in the ischemic kidney due to the fact that the contralateral kidney remained unaffected (Fig. 3A,B). Moreover, significantly more renal fibrosis was observed upon chronic ischemic injury and AAI or CP-induced CKD. These signs of chronic kidney damage were consistent with pathological characteristics on day 30, i.e. increased fibrosis as evidenced by Masson-trichrome (MT) and α-SMA staining (Fig. 3B; data not shown) as well as increased infiltration of F4/80 + macrophages (Fig. 3C). Moreover, ischemic and AAI-treated mice exhibited a significant loss of proximal tubule as evidenced by *lotus tetragonolobus (asparagus pea) lectin* staining compared to the vehicle and CP or FA-treated mice (Fig. 3D). To determine whether these findings are consistent with kidney injury and inflammation, mRNA expression profiling of total kidney tissue from all tested models was carried out (Fig. 4). Ischemic and AAI-treated mice strongly enhanced intrarenal mRNA expression of the kidney damage marker Kim-1 and NGAL. Various pro-inflammatory and pro-fibrotic cytokines including IL-6, TNFα, TGFβ, IL-13 and FSP-1 were increased in renal tissue upon IRI, AA, and CP injury, whereas FA-induced injury was less pronounced (Fig. 4). In addition, genes that influence the functional phenotype of macrophages and therefore regulate tissue remodeling and fibrosis in renal tissue were differently regulated across all animal models. We observed an up-regulation of multiple alternative macrophage-like cytokines such as TGFβ, IL-10 and IL-13 and genes such as IRF4 that are responsible for the switch into this particular macrophage phenotype in IRI, AAI and CP-treated mice. Interestingly, the classically activated macrophage related factor IRF5 did not change in AA-treated mice compared to the control group. However, we observed an increase of IRF4 that suggests a macrophage phenotype switch towards alternatively activated phenotype. Together, expression of genes that are important for the development of pro-inflammatory (IL-6, TNFα, IRF5) and alternative activated (IL-10, IRF4, IL-4, IL-13) macrophages in all selected models increased and their levels were consistent with the high numbers of infiltrating macrophages (Fig. 1). Moreover, some of the models, especially the AA-induced model, showed a stronger pro-fibrotic macrophage phenotype than others.

### AA induced cell death in renal tubular cells and macrophages *in vitro* as well as phenotype of macrophages *in vivo*

**Figure 5:**
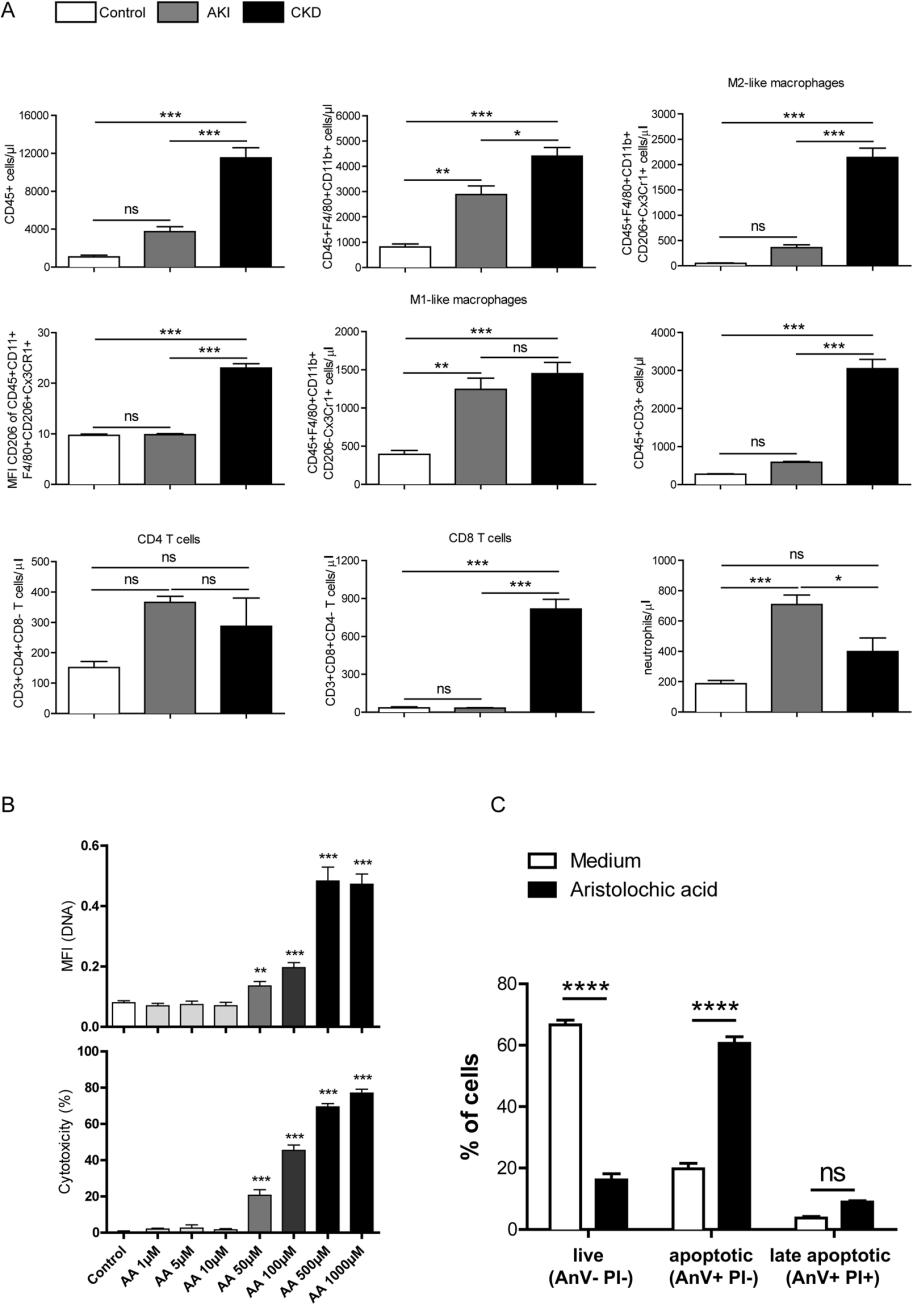
AA-induced modulation of macrophage phenotype *in vivo* and effects on macrophages *in vitro*. (**A**) Flow cytometry analysis of leukocyte/lymphocyte (CD45+) classically activated macrophages (CD45+CD11b+F4/80+CD206−Cx3CR1+), alternatively activated macrophages (CD45+CD11b+F4/80+CD206+Cx3CR1+), CD3+ cells (CD45+CD3+), CD4+ T-cells (CD45+CD3+CD4+CD8−), CD8 + T-cells (CD45+CD3+CD4+CD8+) and neutrophils (CD45+CD11b+Ly6G+) after acute and chronic AAI-induced kidney injury. (**B**) Cell viability of macrophages was assessed by PI staining and LDH assay up to 96 hours in medium supplemented with 2% FCS. Data represents mean values 72 hours after stimulation with indicated AAI concentrations ± SEM of three independent experiments; *p < 0.05 **p < 0.01, ***p < 0.001. (**C**) Macrophages were stimulated with 50 µM AAI for 24 hours and stained with AnnexinV (AnV) and PI. Early apoptotic (AnV+ PI−) and late apoptotic/primary necrotic (AnV+ PI+) macrophages were identified by flow cytometry. Data are expressed as means ± SEM; ****p < 0.0001, AAI versus medium control group.

The *in vivo* AA-induced CKD model showed a moderate acute renal phenotype and typical features of chronic AAN such as tubular damage with atrophy and tubulointerstitial fibrosis as well as a strong macrophage infiltration. First, we investigated the effect of AAI on macrophages phenotype *in vivo*. We performed flow cytometry of kidney myeloid cell suspension after acute and chronic injury and compared them with a control group. We confirmed our previous observations and showed a significant increase of neutrophils and classically activated macrophage in acute injury phase (Fig. 5A). Furthermore, we showed that alternatively activated macrophages significantly increase after chronic kidney injury (Fig. 5A). Additionally we observed significant infiltration of CD45+, CD3+, CD8+ T cells in chronic injury phase that supported the histological analysis (Supplementary Fig. 2). For a better characterization of renal cells and macrophages, we made use of a translational approach and set up *in vitro* experiments. First, we investigated the cytotoxic effect of AAI’s on renal tubular cells and bone marrow-derived macrophages. As shown in (Fig. 5B, Supplementary Fig. 3), the number of dying macrophages (PI + stained cells in red) increased upon stimulation with different concentrations of AA after 24 hours. A quantitative analysis revealed an increase in the mean fluorescence intensity (MFI) of PI, which stains for DNA as well as in the % of cell cytotoxicity as determined by LDH assay (Fig. 5B). This was also the case for renal tubular cells that were stimulated with various concentrations of AA compared to the control for 24 hours (Supplementary Fig. 3). The data indicate that AA induced cell death in renal tubular cells and bone marrow-derived macrophages in a dose-dependent manner. We identified 10 µM and 50 µM AAI as the optimal concentrations for further *in vitro* experiments. To characterize the form of cell death induced by AAI in more detail, bone marrow-derived macrophages were cultured in the presence of 50 µM AAI for 24 hours and flow cytometry analysis for AnnexinV (AnV) and PI carried out. As shown in Fig. 5C, approximately 60% of macrophages died via early apoptosis (AnV+ PI−) and 10% of cells were identified as late apoptotic/primary necrotic (AnV+ PI+) upon AA treatment (Fig. 5C). Thus AAI induced primarily apoptosis in macrophages.

### AA exposure modulates macrophage phenotype *in vitro*

**Figure 6:**
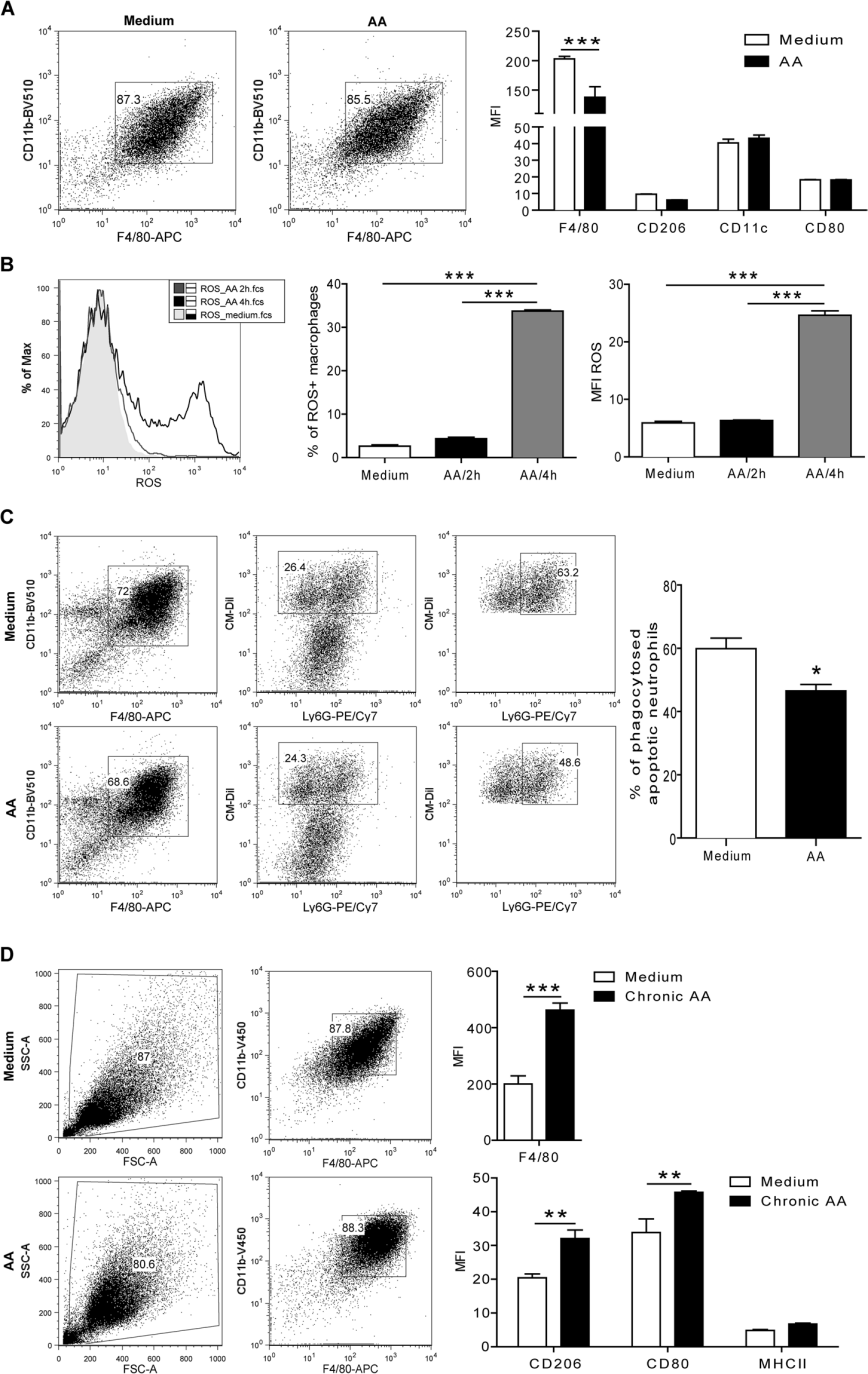
AA-induced modulation of macrophage phenotypes *in vitro*. (**A**) Flow cytometry analysis of acute exposed macrophages (F4/80+CD11b+) with AAI demonstrated by the mean fluorescent intensity (MFI) of the surface marker F4/80, CD206, CD11c and CD80 compared to untreated macrophages (medium). Data are expressed as means ± SEM (AAI versus medium control group); ***p < 0.001. (**B**) Intracellular ROS production of AAI-stimulated macrophages for 2 and 4 hours indicated by the % of ROS+ macrophages and the MFI of ROS compared to untreated cells (medium). (**C**) AAI-treated and untreated macrophages (F4/80+CD11b+) were co-incubated with apoptotic neutrophils (AnnexinV+CM-Dil+) and the % of phagocytosed apoptotic neutrophils by macrophages (F4/80+CD11b+ AnnexinV+CM-Dil+Ly6G+) determined by flow cytometry. (**D**) Chronic exposure of macrophages with AAI or untreated macrophages (F4/80+CD11b+) were characterized for the expression levels of F4/80, CD206, CD80 and MHCII as MFI by flow cytometry. Data are presented as the means ± SEM (n = 6–10). *p < 0.05, **p < 0.01, ***p < 0.001.

To investigate the effect of acute and chronic AA-exposure on the functional role of macrophages in controlled *in vitro* environment, bone marrow-derived macrophages were stimulated with 50 µM AAI for 48 hours. Flow cytometry analysis revealed that AA-treated macrophages downregulated the macrophage surface marker F4/80 but no difference was observed on the expression of CD206, CD11c and the activation marker CD80 compared to medium (Fig. 6A). Intracellular ROS production upon AA treatment significantly increased in macrophages as indicated by an increase in the % of ROS+ macrophages and MFI of ROS (Fig. 6B). On the other hand, the ability of AA-treated macrophages to phagocytose CM-Dil+ Ly6G+ apoptotic neutrophils decreased compared to untreated macrophages (Fig. 6C, Supplementary Fig. 3). This indicated that an acute AA exposure induced ROS production but decreased the phagocytic capacity of macrophages, therefore driving a more pro-inflammatory macrophage phenotype. Our *in vivo* data demonstrated that macrophages were present in high numbers in the kidneys of AA-treated mice on day 30 and that these macrophages might display a more anti-inflammatory-like macrophage phenotype as indicated by increased IRF4 gene expression. To investigate whether we can drive an alternative macrophage phenotype under chronic AAI exposure also *in vitro*, macrophages were cultured in the presence of AAI for 6 days as described in material and methods, and flow cytometry was carried out. Chronic AA treatment resulted in a significant increased expression of the macrophage surface marker F4/80 and the alternative macrophage marker CD206 as well as the activation marker CD80 compared to medium alone (Fig. 6D). No difference was observed in MHCII expression. The data indicated that chronic AA exposure of macrophages can be used as an *in vitro* model to further characterize the function of alternatively activated macrophages. Together, we showed that AAI is an appropriate stimulus to study detailed questions associated with macrophage phenotypes *in vitro*.

## Discussion

Researchers use experimental renal injury models to uncover therapeutic targets and to better understand the pathology underlying acute and chronic renal failure. However, the variability of protocols among laboratories limits the comparability between studies and abates translational research^36,37^. Our study was designed to compare in detail four different mouse models and modify them for better evaluation of both the acute and the chronic renal injury phase. For instance, to investigate cisplatin-induced acute and chronic kidney damage the dosage of cisplatin was decreased in order to improve the survival rate and enable the regeneration. Similarly, the single dosage of AAI per kg was reduced. Six consecutive injections on every alternate day were carried out to elicit intense and sustained inflammation. We observed acute renal damage and robust infiltration of macrophages, especially in ischemic injury and AAI-treated animals. Moreover, all tested models displayed some features of chronic kidney disease associated with renal fibrosis. The effects of AAI on renal epithelial cells have already been investigated *in vitro* and revealed cytotoxic properties on membrane integrity and affecting cellular viability^18^. Our data showed that the progressive accumulation of macrophages was most prominent in AAI-treated as well as ischemic mice and that these two models are appropriate to study macrophage phenotypes in kidney injury. Both models induce severe acute tubular necrosis followed by inflammation and fibrosis. Ischemia plays a crucial trigger of acute kidney injury in the human population and is a risk factor of developing subsequent CKD^7^. However, the long-term consequences of bilateral renal ischemia cannot be evaluated due to severe phenotype associated with increased mortality. Our data showed that a unilateral IRI does not change renal parameter. Moreover, the outcome may be proportional not only to the period of ischemia, but also to temperature during the operative procedure^38^. The variations in protocols can make comparative studies of therapeutic interventions difficult. Unlike ischemia, AAI-induced injury leads to increased BUN and creatinine serum levels 30 days after the first injection of AAI (5 mg/kg). With the protocol described in this study, we observed a high disease incidence and a consistent, reliable disease course without episodes of mortality within a group. Previous investigations showed that a single i.p. dose of 10 mg/kg AAI leads to AKI followed by death within 14 days^39^. In addition, we showed that the AAI-induced model might be of interest for studying macrophage phenotype-related contributions due to the high accumulation of renal macrophages. The persistence of interstitial inflammation during the chronic phase was associated with increased kidney injury and F4/80+ macrophages. Among immune cells that infiltrate the kidney upon acute injury, monocytes and macrophages might be the main regulators of inflammation, tissue remodeling and fibrosis^40^. Furthermore, unlike ischemic injury, the AAI-induced injury model can be easily mimicked *in vitro*. We confirmed that acute AAI exposure can modulate oxidative stress processes by enhancing ROS production *in vitro*, and at the same time decreasing the ability of macrophages to phagocytose apoptotic neutrophils. This is interesting and would suggest that acute AAI exposure drives a pro-inflammatory macrophage phenotype with decreased phagocytic abilities compared to alternatively activated macrophages^41,42^. Macrophage-associated ROS production is an important feature of many physiological processes, particularly in host’s innate immunity responses^43,44^. However, the release of ROS includes not only nitric oxide but also superoxide-anion, hydrogen peroxide, hydroxyl radical etc. that are associated with mitochondrial respiration^45^. Zhang *et al*. described that ROS production is important in activated M2-differentiated but not in M1-like macrophages^46^. Nevertheless, their role in macrophage polarization is yet unclear. Using an approach of inducing acute and chronic AAI-associated injury *in vivo* and *in vitro*, we demonstrated the immunomodulatory capacity of AAI in modulating the functional phenotype of macrophages. Importantly, AAI is known to affect the anemia caused by eryptosis and impaired blood circulation^47–49^. Although, the detailed molecular mechanisms responsible for AA-induced anemia remain unclear, released hemoglobin that undergoes glomerular filtration may significantly affect the renal tubules damage^50^. Our study focused on the influence of AAI on macrophage phenotype and local renal phenomenon. However, considering of systemic effects of AAI is important when applying the model.

Animal models will remain pivotal for translational research and require precise experimental design in order to obtain valid information about pathophysiological processes. Therefore, appropriate cell culture models should be applicable for conducting preliminary experiments to reduce the amount of animals used for *in vivo* studies. Here, we characterized the model that yields a consistent and robust renal pathology shown by changes in serum BUN and creatinine levels without increased mortality. Moreover, we studied in detail the role of AAI in macrophage biology.

## Electronic supplementary material

Supplementary Information
