# Supplementary material for: Aristolochic acid I determine the phenotype and activation of macrophages in acute and chronic kidney disease
Source: Sci Rep. 2018 Aug 15;8:12169. doi: 10.1038/s41598-018-30628-x (PMC6093867; doi:10.1038/s41598-018-30628-x)
Supplement: Supplementary file 1 — Supplementary Information [file 41598_2018_30628_MOESM1_ESM.pdf]

Aristolochic acid I determine the phenotype and activation of macrophages in acute and chronic kidney disease

Mohsen Honarpisheh[1]*, Orestes Foresto-Neto[1]*, Stefanie Steiger[1], Franziska Kraft[1], Paulina Koehler[1], Ekaterina von Rauchhaupt[1], Jan Potempa[2], Karina Adamowicz[2], Joanna Koziel[2], Maciej Lech[1,2]

[1] Klinikum der Ludwig-Maximilians-Universität München, Medizinische Klinik und Poliklinik IV, Department of Nephrology, LMU Munich, Germany

[2] Departments of Microbiology, Faculty of Biochemistry, Biophysics and Biotechnology, Jagiellonian University, Krakow, Poland;

* Equal contribution

Supplementary figure 1

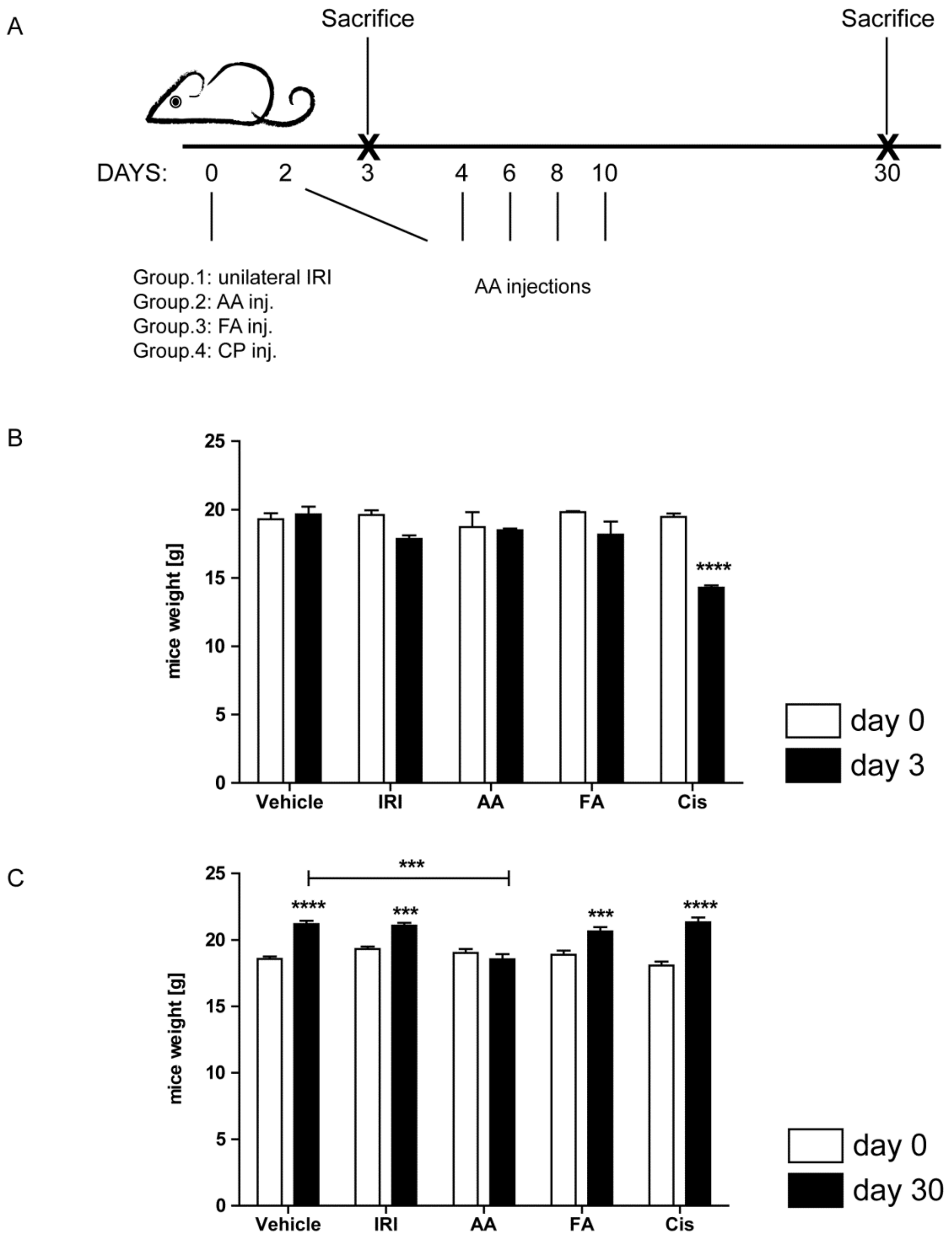

Supplementary figure 1. (A) Schematic outline of the experimental procedures. IRI, ischemia-reperfusion injury, AA, aristolochic acid; CP, cisplatin; FA, folic acid; (B) Daily changes in body weight during the induction of acute kidney disease. Data are presented as the means ± SEM (n=6-10); **** p<0.0001; (C) Changes in body weight during the induction of chronic kidney disease. Data are presented as the means ± SEM (n=6-10). *** p<0.001, **** p<0.0001.

Supplementary figure 2

A

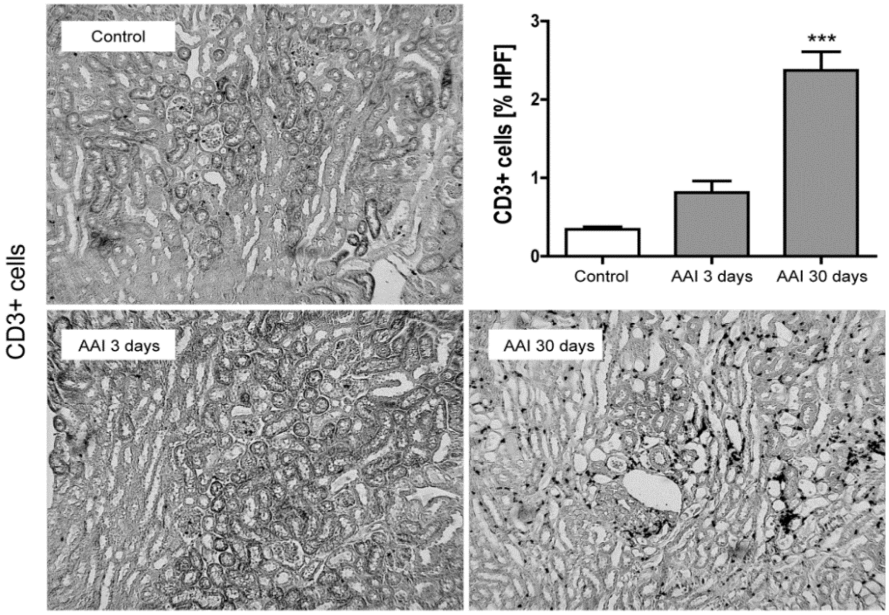

B

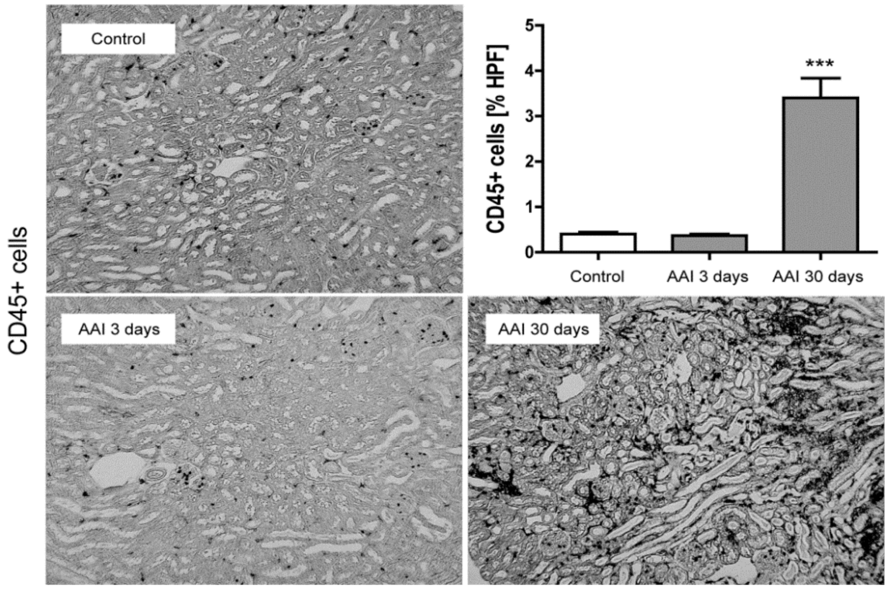

Supplementary figure 2. Infiltration of CD3+ and CD45+ cells in AKI and CKD upon AAI. (A) Wild type mice underwent intervention (AAI) and kidneys were harvested 3 or 30 days later. Renal sections were stained for CD3 antigen (B), or CD45; Images illustrate representative sections. The indices for cell infiltration were determined by quantitative morphometry as described in the methods section. Values represent means ± SEM from 15 high power fields of investigated kidneys from 6-10 mice in each group. Data are expressed as means ± SEM. *** $p<0.001$ versus control animal group.

Supplementary figure 3

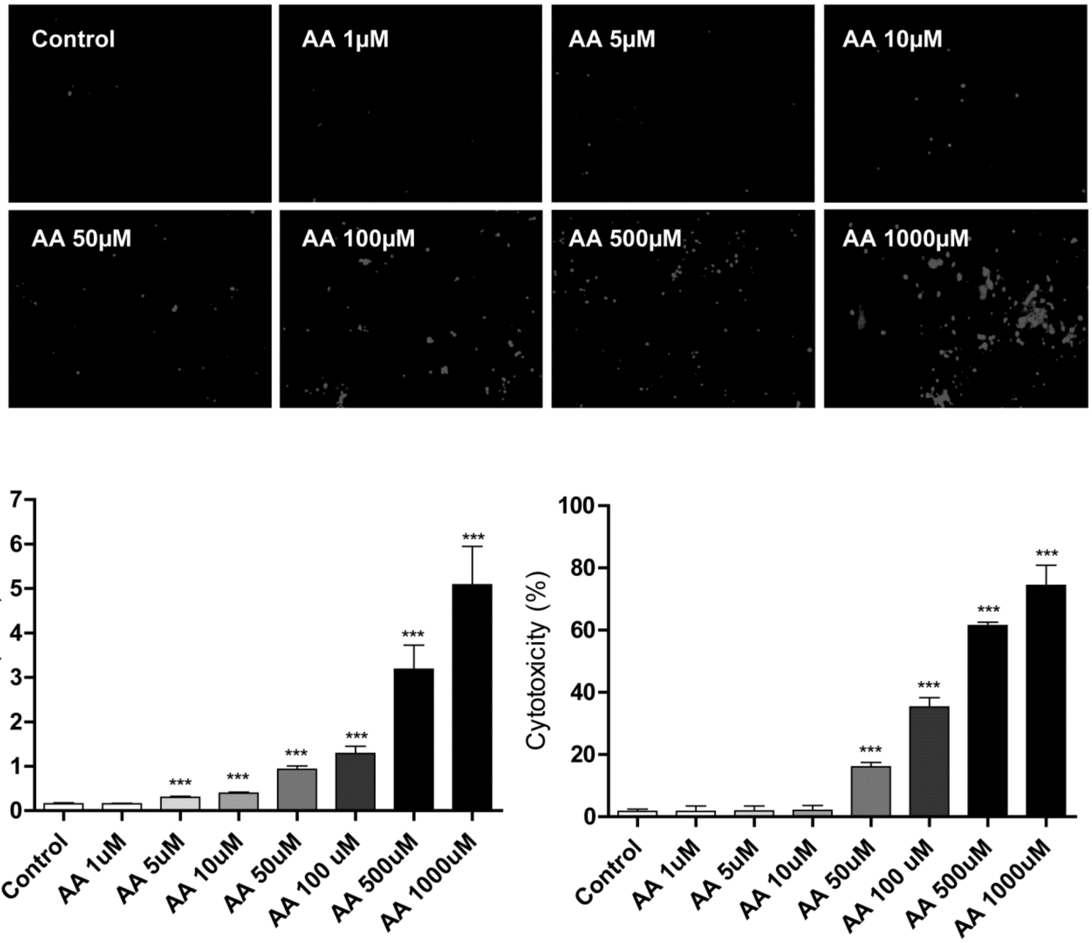

Supplementary figure 3. AA-dependent *in vitro* effects on proximal tubular cells Cell viability of macrophages were assessed by PI staining and LDH assay up to 96 hours in medium supplemented with 2% FCS. Data represents mean values 72 hours after stimulation with indicated AAI concentrations ± SEM of three independent experiments; *** $p<0.001$.

Supplementary figure 4

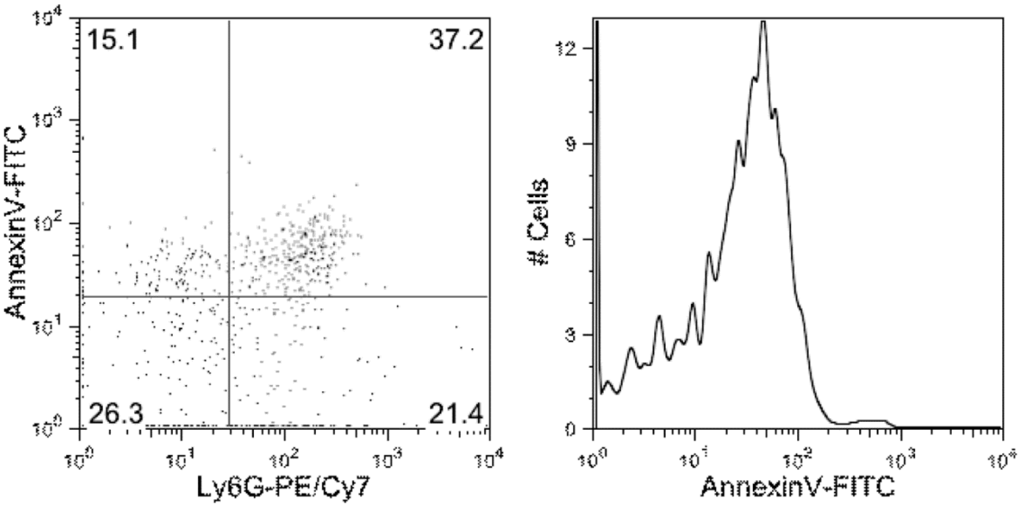

Supplementary figure 4. Induction of apoptosis in neutrophils. Cells were isolated from the air pouch fluid and stained with the cell tracker CM-Dil fluorescent dye. Apoptosis of CM-Dil stained neutrophils was induced with TNFα and phorbol myristate acetate, and apoptotic neutrophils identified as AnnexinV+ CM-Dil+ Ly6G+ by flow cytometry.
